# ONE STAGE BILATERAL TOTAL HIP REPLACEMENT

**DOI:** 10.1590/1413-785220243204e278347

**Published:** 2024-10-07

**Authors:** Fabio Stucchi Devito, Fabio Stucchi Devito, Eduardo Dias Devito, Cristiane Bonvicine

**Affiliations:** 1. Faculdade de Medicina de Sao Jose do Rio Preto FAMERP, Departamento de Ortopedia e Traumatologia, Sao Jose do Rio Preto, SP, Brazil; 2. Hospital do Servidor Publico Estadual, Departamento de Ortopedia, Sao Paulo, SP, Brazil; 3. Faculdade de Medicina de Sao Jose do Rio Preto FAMERP, São Jose do Rio Preto, SP, Brazil

**Keywords:** Total Hip Arthroplasty, Surgery, Hip, Artroplastia Total De Quadril, Cirurgia, Quadril

## Abstract

**Introduction:**

One-stage bilateral total hip replacement has gained popularity due to its advantages, which include its lower cost, anesthetic time, hospitalization, and recovery.

**Objective:**

to show the clinical result of one-stage bilateral total hip replacement.

**Methodology:**

A case series of patients who underwent one-stage bilateral total hip arthroplasty. The medical records of 100 patients were evaluated from 2001 to 2022. The posterolateral route was chosen for the procedures. Of the 100 replaced prostheses, 85% were hybrid and 15 were cemented. Procedures averaged 180 minutes in length.

**Results:**

The average length of stay totaled three days. No deaths occurred in the 100 evaluated patients. Complications showed 1% rate of venous and pulmonary thromboembolism, one case of late dislocation (after three months. It was twice reduced and later revised), five cases of hematoma (5%. They were drained on the third postoperative day. Moreover, two occurred in both hips).

**Conclusion:**

One-stage bilateral total hip replacement has advantages but it must be performed on carefully selected patients and by a qualified team. ***Evidence level IV, Case reports.***

## RESUMO

**Introdução:**

O método que realiza a prótese total de quadril bilateral em um único tempo operatório (ATQB) tem ganhado popularidade por suas vantagens. Denntre essas, destacam-se menor custo, tempo anestésico reduzido, e menor período de internação e recuperação.

**Objetivo:**

Demonstrar o resultado clínico da ATQB em um único tempo.

**Metodologia:**

Estudo retrospectivo pacientes submetidos a Artroplastia total de quadril bilateral em um único tempo. Foram avaliados prontuários de 100 pacientes, durante o período de 2001 a 2022. A via escolhida foi a póstero-lateral. Das 100 próteses realizadas, 85% foram híbridas e 15 cimentadas. O tempo cirúrgico teve, em média, 180 minutos.

**Resultados:**

O tempo médio de internação foi de 3 dias. Não houve mortes entre os 100 pacientes avaliados; quanto às complicações, a taxa de tromboembolismo venoso e pulmonar foi de 1%, e foi relatado 1 caso de luxação tardia (após 3 meses), reduzida duas vezes e com posterior revisão. Houve cinco casos de hematoma, totalizando 5%; os mesmos foram drenados no 3º dia pós-operatório; desses, 2 aconteceram bilateralmente e 1 unilateralmente.

**Conclusão:**

*A ATQB em um único e cirúrgico tempo apresenta vantagens, mas deve ser realizada em pacientes cuidadosamente selecionados e por uma equipe capacitada. **Nível de evidência IV, Série de casos.***

## INTRODUCTION

 Total hip replacement (THR) is a surgical procedure that is widely used around the world to treat conditions such as osteoarthritis and other hip joint diseases. ^1^ Recent years have seen a significant increase in patients requiring bilateral hip replacement, forcing surgeons to make a crucial decision: one- or two-stage surgery. 

 The method for bilateral total hip prosthesis involving a one-stage operative time (known as step 1) has gained popularity for its advantages, including its lower cost, ^2^^,^^3^ anesthetic time, hospital stay ^4^ , and rehabilitation, in addition to better limb length control. ^3^

 Bilateral single-stage prosthesis was first described by Charnley in 1971, showing excellent results since then. ^1^

 However, several factors influence the choice between one- or two-stage surgery, including patients’ age and health and surgeons’ experience. ^3^ This study aims to show the clinical outcomes of one-stage bilateral THR. 

## MATERIALS AND METHODS

This is a retrospective observational study in which patients underwent one-stage bilateral total hip arthroplasty in the service. Medical records of 100 people were evaluated from 2001 to 2022. The ages of the 21 women and 79 men ranged from 17 to 76 years.

The American Society of Anesthesiologists (ASA) physical status classification categorized patients into ASA 1 or 2.

Regarding the used route, all cases involved the posterolateral Kocher-Langenbeck route to the acetabulum.

Of the 100 prostheses performed, 85% were hybrid (non-cemented acetabular and cemented femoral components) and 15 were cemented (acetabulum and femur).

Surgeries lasted from 120 to 240 minutes, averaging 180 minutes.

One gram of tranexamic acid was intraoperatively used to reduce blood loss in 78 cases, and blood transfusion ranged from zero to two bags of packed red blood cells, averaging one transfused bag. All patients needed a pneumatic pump foot for their lower limbs during hospitalization to prevent deep vein thrombosis (DVT) and pulmonary thromboembolism (PTE).

All patients started walking on the first postoperative day, assisted by physical therapy and a gait aid. They also performed isometric and metabolic active exercises.

All cases included a suction drain that was removed 24 hours after the procedure.

**Table 1.** Casuistry table

Age	17 to 76 years
Female	21
Male	79
Hybrid prosthesis	85
Cemented prostheses	15
Surgical time	120 to 240 minutes
Length of hospital stay	From 2 to 5 days

## RESULTS

Mean hospital stay totaled three days, ranging from two to five days.

Of the 100 evaluated patients, mortality rate totaled 0%.

Regarding thromboembolic events, the rate of DVT and PTE totaled 0.1%.

No patient showed acute dislocation, but one evinced late dislocation (after three months), which was reduced twice and necessitated later revision.

This study included five cases of hematoma (two bilateral and three unilateral ones, 5%) that were drained on the third postoperative day.

**Table 2.** Results

		Rate	
Mortality rate	0 cases	0%	
DVT	1 case	1%	
PTE	1 case	1%	
Acute dislocation	0 cases	0%	
Delayed Dislocation	1 case	1%	Two reductions and subsequent revision
Hematoma	5 cases	5%	Drained on the third post-op day; two bilaterally and three unilaterally drained.

## DISCUSSION

 Bilateral total hip replacement surgery performed simultaneously under the same anesthesia (step 1) offer excellent results, with advantages such as lower cost, ^2^^,^^3^ anesthetic time, hospital stay, ^4^ and rehabilitation and better control of limb length. ^3^

 This study performed Doppler ultrasound of the lower limbs and pulmonary computed tomography in patients with symptoms, finding only one case of PTE (1%). This data is similar to that in the literature, as in the 2018 study by Charity et al., which reported only two cases of PTE in 319 prostheses (0.62%). Jaffe and Charnley, in a series of 50 cases of bilateral THR in 1 step, showed two cases of pulmonary complications (4%). ^3^

 The literature shows no statistical differences in pulmonary complications between one- and two-stage bilateral THR. ^5^^,^^6^^,^^7^^,^^8^ . However, this technique requires care and adequate selection of patients, especially those with lung problems and lower right ventricular reserve. ^9^

 Regarding acute dislocation, we found no cases. As reported, only one patient had late dislocation, possibly because, when the bilateral hip prosthesis is performed in a one stage, anatomical reconstruction, gluteus medius strength, and the positioning of the prosthesis components are easier to replicate on the other side to be immediately operated. Tsiridis et al, in 2008, reported no statistical difference on the risk of acute dislocation between one- and two-stage THR. ^5^ Huang et al, in 2019, in the series of 16,758 bilateral THR, reinforce the absence of statistical differences in hip dislocation, ^7^ finding lower rates in other one-stage studies. ^10^

This study found no mortality. In 2018, Charity et al. reported a 0.3% six-month mortality rate. English registries show an incidence of 0.29% in unilateral THR, evincing the similarity between step 1 and 2 incidences.

 Huang et al reinforces the absence of significant differences in mortality and cardiovascular and/or infection problems. ^7^ This study found no acute and/or sub-acute infection. The five patients with hematoma underwent drainage on the third postoperative day, collecting the hematoma culture and sending it to a laboratory for analysis (which tested negative for infection). In our view, a more invasive measure in the immediate postoperative period, with drainage and surgical cleaning, helps to prevent infections. In these cases that required drainage, we resumed broad-spectrum antibiotic therapy until the results of the cultures were obtained. 

 Micicoi et al, in 2004-2018, reported a revision rate of 2.3% for bilateral total hip replacements in a single procedure, compared to 4.1% for unilateral prostheses. Ramezani et al., on the other hand, found no significant difference between the two groups. ^10^

The literature reinforces the data in our series: low revision rates and even lower than in two-stage periods. This suggests that the choice between step 1 and step 2 may depend on the proper selection of patients and surgeons’ experience.

 Partridge et al., in 2019, report the need for adequate team experience, the procedure taking place in large centers, and patient being adequately selected (involving age and comorbidity assessments). ^11^ In our view, the choice of the patient, the training of the team, the short surgical time (180 minutes for both sides), usual access route, and a surgeon used to the procedure contribute to the good result of this type of procedure. Also, the reproducibility of the previous surgery on the following side is much better, as is the adequacy of the offset to the length of the limbs. 

 The techniques and protocols physical therapy use to treat single-stage total hip arthroplasty vary (as in two-stage total hip arthroplasty) but have the advantage of shorter rehabilitation time. They have important clinical efficacy proven in the literature. Patients’ results after single-stage THR, such as better functionality, muscle strength, and range of motion, corroborate the results in the literature. In general, active exercises for the hip periarticular muscles provided an important functional prognosis. ^12^^,^^13^^,^^14^

## CONCLUSION

Bilateral total hip arthroplasty should be performed in a single surgical procedure for carefully selected ASA 1 or 2 patients and by a trained surgical team. Our series chose the posterolateral access, but surgeons may choose other routes, depending on their preference. Rehabilitation is similar to two-stage surgery, with the advantage of a shorter recovery time.

## References

[1] Stavrakis A. I., SooHoo N. F., Lieberman JR (2015). Bilateral Total Hip Arthroplasty has Similar Complication Rates to Unilateral Total Hip Arthroplasty. J Arthroplasty.

[2] Reuben J. D., Meyers S. J., Cox D. D., Elliott M., Watson M., Shim S. D. (1998). Cost comparison between bilateral simultaneous, staged, and unilateral total joint arthroplasty. J Arthroplasty.

[3] Jaffe WL, Charnley J (1971). Bilateral Charnley low-friction arthroplasty as a single operative procedure. A report of fifty cases. Bull Hosp Joint Dis.

[4] Saito S., Tokuhashi Y., Ishii T., Mori S., Hosaka K. (2010). One- versus two-stage bilateral total hip arthroplasty. Orthopedics.

[5] Tsiridis E., Pavlou G., Charity J., Tsiridis E., Gie G., West R. (2008). The safety and efficacy of bilateral simultaneous total hip replacement: an analysis of 2063 cases. J Bone Joint Surg Br.

[6] Micicoi G., Dompsure R. B., Micicoi L., Tran L., Carles M., Boileau P., Trojani C. (2020). One-stage bilateral total hip arthroplasty versus unilateral total hip arthroplasty: a retrospective case-matched study. Orthop Traumatol Surg Res.

[7] Huang L., Xu T., Li P., Xu Y., Xia L., Zhao Z. (2019). Comparison of mortality and complications between bilateral simultaneous and staged total hip arthroplasty: a systematic review and meta-analysis. Medicine (Baltimore).

[8] Shao H., Chen C.-L., Maltenfort M. G., Restrepo C., Rothman R. H., Chen A. F. (2017). Bilateral Total Hip Arthroplasty: One-stage or Two-stage? A Meta-analysis. J Arthroplasty.

[9] Memtsoudis S. G., Salvati E. A., Go G., Ma Y., Sharrock N. E. (2010). Perioperative pulmonary circulatory changes during bilateral total hip arthroplasty under regional anesthesia. Reg Anesth Pain Med.

[10] Ramezani A., Ghaseminejad Raeini A., Sharafi A., Sheikhvatan M., Mortazavi S. M. J., Shafiei S. H. (2022). Simultaneous versus staged bilateral total hip arthroplasty: a systematic review and meta-analysis. J Orthop Surg Res.

[11] Partridge T. C. J., Charity J. A. F., Sandiford N. A., Baker P. N., Reed M. R., Jameson S. S. (2020). Simultaneous or Staged Bilateral Total Hip Arthroplasty? An Analysis of Complications in 14,460 Patients Using National Data. J Arthroplasty.

[12] Budib M. B., Hashiguchi M. M., Oliveira-Junior S. A., Martinez P. F. (2020). Influence of physical rehabilitation on functional aspects in individuals submitted to total hip arthroplasty: a systematic review. Rev Bras Geratr Gerontol.

[13] Cezarino L., Vieira W., Silva J., Silva-Filho E., Souza F., Scattone R. (2019). Gait and functionality following unilateral and bilateral hip replacement. Fisioter Mov.

[14] Charity J., Wyatt M. C., Jameson S., Whitehouse S. L., Wilson M. J., Gie G. A. (2019). Is single-anaesthetic bilateral total hip replacement using cemented stems safe and appropriate? A review of four decades of practice. Hip Int.

